# Optical Aptamer-Based Cytokine Nanosensor Detects Macrophage Activation by Bacterial Toxins

**DOI:** 10.1021/acssensors.4c00887

**Published:** 2024-06-27

**Authors:** Amelia K. Ryan, Syeda Rahman, Ryan M. Williams

**Affiliations:** † Department of Biomedical Engineering, The City College of New York, New York, New York 10031, United States; ‡ PhD Program in Chemistry, Graduate Center, City University of New York, New York, New York 10016, United States

**Keywords:** IL-6, DNA aptamer, inflammation, nanocarbon, SWCNT, fluorescence

## Abstract

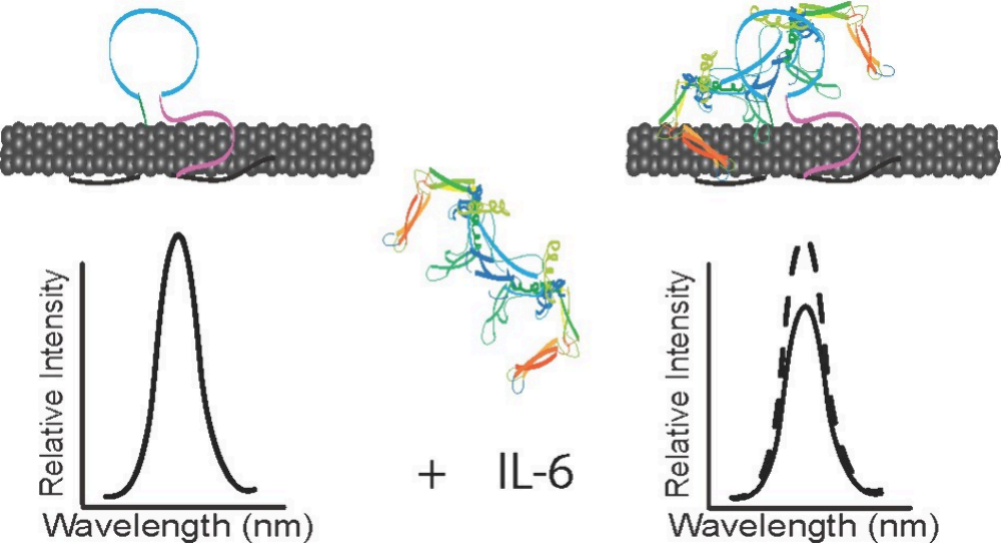

Overactive or dysregulated cytokine expression is a hallmark of many acute and chronic inflammatory diseases. This is true for acute or chronic infections, neurodegenerative diseases, autoimmune diseases, cardiovascular diseases, cancer, and others. Cytokines such as interleukin-6 (IL-6) are known therapeutic targets and biomarkers for such inflammatory diseases. Platforms for cytokine detection are, therefore, desirable tools for both research and clinical applications. Single-walled carbon nanotubes (SWCNT) are versatile nanomaterials with near-infrared fluorescence that can serve as transducers for optical sensors. When functionalized with an analyte-specific recognition element, SWCNT emission may become sensitive and selective toward the desired target. SWCNT-aptamer sensors are easily assembled, inexpensive, and biocompatible. In this work, we introduced a nanosensor design based on SWCNT and a DNA aptamer specific to IL-6. We first evaluated several SWCNT-aptamer constructs based on this simple direct complexation method, wherein the aptamer both solubilizes the SWCNT and confers sensitivity to IL-6. The sensor limit of detection, 105 ng/mL, lies in the relevant range for pathological IL-6 levels. Upon investigation of sensor kinetics, we found rapid response within seconds of antigen addition which continued over the course of 3 h. We found that this sensor construct is stable and the aptamer is not displaced from the nanotube surface during IL-6 detection. Finally, we investigated the ability of this sensor construct to detect macrophage activation caused by bacterial lipopolysaccharides (LPS) in an in vitro model of disease, finding rapid and sensitive detection of macrophage-expressed IL-6. We are confident that further development of this sensor will have novel implications for diagnosis of acute and chronic inflammatory diseases, in addition to contributing to the understanding of the role of cytokines in these diseases.

Inflammatory diseases, including neurodegenerative diseases, autoimmune diseases, cardiovascular disease, and cancer, among others, are characterized by chronic inflammation which promotes disease progression.^1,2^ Inflammatory cytokines are key initiators and drivers of these diseases. Cytokines such as interleukin-6 (IL-6), interleukin-1 beta (IL-1β), interleukin-8 (IL-8), and tumor necrosis factor-α (TNF-α) are known therapeutic targets and biomarkers for these inflammatory diseases.^3−5^ Platforms for cytokine detection are therefore desirable tools for both research and clinical applications.

Cytokines are small, secreted proteins (<40 kDa) which are produced by many cell types to regulate and influence immune response.^2^ They modulate both acute and chronic inflammation via a complex network of autocrine, paracrine, and endocrine interactions. Various cell populations can produce the same cytokine, and the effects of each cytokine are pleiotropic. Different cytokines may have the same impact, suggesting redundancy, or cause a synergistic effect.^4,6,7^ Cytokines can serve as predictive, diagnostic, or prognostic biomarkers for inflammatory diseases. However, their precise role in the disease is not always clearly defined. In complex disease states, it can be unclear whether dysregulated cytokine signaling is a cause or result of disease or both. The heterogeneity of inflammatory processes in disease states presents further challenges. For example, various studies of Alzheimer’s disease have shown increased, decreased, and unchanged levels of the same cytokine in cerebrospinal fluid.^8,9^ These are relevant unanswered questions for researchers investigating inflammatory disease mechanisms and responses to therapeutics.

Conventional methods for cytokine detection, such as immunoassays and mass spectrometry, require expert personnel, expensive equipment, and long incubation periods. These techniques are therefore not suitable for continuous monitoring or for rapid point-of-care diagnosis.^10^ Nanosensors are powerful detection tools that hold advantages over conventional methods. They have shown great promise in point-of-care devices, personalized medicine, and accessible techniques for early diagnostics.^11−13^ Nanosensors that utilize optical signal transduction methods are particularly attractive due to their potential for high-resolution imaging and minimally invasive in vivo sensing.^14^

Single-walled carbon nanotubes (SWCNTs) are versatile materials that can be functionalized to detect a variety of clinically relevant molecules.^15,16^ They emit near-infrared (NIR) fluorescence, which is ideal for biological imaging applications due to high tissue penetration depth and minimal autofluorescence of biological tissues in this region.^16−18^ SWCNT fluorescence is stable over time and not subject to photobleaching, providing a substantial advantage over conventional fluorophores.^17,19^ The unique optical properties of SWCNT are due to their structure, which can be visualized as a single sheet of graphene lattice rolled into a cylinder. The angle and diameter at which the sheet is rolled determines the chirality of the nanotube, categorized by an (*n*,*m*) index.^15,20−22^ Each SWCNT chirality exhibits a distinct absorption and fluorescence emission band, which can be modulated by changes in the surrounding environment.^23^ SWCNTs are often functionalized with a molecular recognition probe to confer analyte specificity to these fluorescence modulations.

Approaches for SWCNT-based sensor synthesis often include screening a library of synthetic polymers, computational analytics, and rational design with a molecular recognition probe.^18^ While screening approaches have enabled the discovery of new nanosensors with tunable properties, this may be time-consuming, labor-intensive, and open-ended.^24^ Computational approaches have begun to emerge with substantial benefits; however, large sets of samples and data are required.^25^ Alternatively, rational sensor design often exploits previously validated biological recognition elements with proven affinity for a specific antigen, such as an antibody or aptamer.^24,26^

In this work, we designed a novel DNA aptamer-SWCNT optical sensor construct to detect IL-6.^27^ There have been previous studies that developed optical SWCNT-based sensors for IL-6, each with suitable sensitivity and selectivity.^28−30^ However, one such sensor relied on a rational design with a commercial antibody as the recognition element, which may cause issues related to reproducibility/batch variation and large size/stability.^31−33^ Another sensor relied on a synthetic polymer library screen, which may not be widely available.^28^ We chose to design an aptamer-based sensor for IL-6 as their synthesis is highly reproducible and cost-effective.^34−37^ We took advantage of a previously reported aptamer with demonstrated affinity and function for IL-6.^38^ Previous SWCNT-aptamer sensors have proven to be highly effective in detecting their target of interest.^34,35,39−41^ Those studies largely explored multistep methods for SWCNT-aptamer complexion, though here we explored a direct one-step process wherein the aptamer both solubilizes the SWCNT and confers analyte specificity. We investigated several direct-complexation designs as well as the kinetics, range, and stability of this sensor and its function in complex biological media. Finally, we evaluated the sensor’s function using an in vitro disease model with activated macrophages.

## Methods

### Preparation of ssDNA-SWCNT

ssDNA sequences (Integrated DNA Technologies; Coralville, IA), which were previously obtained via in vitro selection (SELEX, or the Selective Evolution of Ligands by Exponential Enrichment) for their ability to bind to IL-6, were used to impart specificity for IL-6 in our sensor design (Table 1).^38^ The sequence 31Apt was the full-length aptamer obtained from prior studies, while 15Apt was a truncated version reported in that study. (GT)_15_ was used as a control, non-IL-6-binding ssDNA sequence of similar size, as it has previously been used extensively to stably encapsulate the SWCNT. Further, we evaluated a (GT)_15_-31Apt hybrid sequence. We also used fluorescently labeled versions (Cy3 and Cy5, respectively) of (GT)_15_ and 31Apt to evaluate their binding and stability.

**Table 1:** ssDNA Sequences Used in Sensor Testing

name	ssDNA sequence
(GT)_15_ control	5′-GTGTGTGTGTGTGTGTGTGTGTGTGTGTGT-3′
15Apt	5′-GGTGGCAGGAGGACTA-3′
31Apt	5′-GGTGGCAGGAGGACTATTTATTTGCTTTTCT-3′
(GT)_15_+31Apt	5′-TGTGTGTGTGTGTGTGTGTGTGTGTGTGTGGTGGCAGGAGGACT ATTTATTTGCTTTTCT-3′
(GT)_15_+Cy3	5′-GTGTGTGTGTGTGTGTGTGTGTGTGTGTGT/3Cy3Sp/-3′
31Apt+Cy5	5′-GGTGGCAGGAGGACTATTTATTTGCTTTTCT/3Cy5Sp/-3′

SWCNT-ssDNA suspensions were prepared as previously described.^10,42−45^ Briefly, HiPCO-prepared SWCNT (NanoIntegris Technologies; Boisbriand, QC) and DNA in a 1:2 mass ratio were suspended in 1× PBS. Samples were sonicated on ice at 40% amplitude for 1 h by a 120 W ultrasonicator with a 1/8″ microtip probe (Fisher Scientific; Hampton, NH). Sonicated suspensions were ultracentrifuged at 58,000*g* for 1 h using an Optima Max-XP Ultracentrifuge (Beckman Coulter; Brea, CA). The top 75% of the suspension was collected. Samples were stored at 4 °C for up to 14 days. Within 24 h of use, samples were filtered to remove free DNA with a 100 kDa Amicon centrifugal filter (Sigma-Aldrich; St. Louis, MO) for 15 min at 14,000*g*. The solution retained in the filter was suspended in fresh 1× PBS.

### UV–Vis Absorbance and Concentration Measurement

After filtration, SWCNT-ssDNA samples were subjected to absorbance measurements from 300 to 1100 nm using a V-730 UV–vis Spectrophotometer (Jasco; Easton, MD). SWCNT-ssDNA solutions were diluted in 1× PBS to achieve absorbance values <0.5. As in prior works,^10,46−48^ the concentration of SWCNT was calculated using the absorbance value at the local minimum near 630 nm (extinction coefficient = 0.036 L/mg·cm).

### Near-Infrared Fluorescence Emission Measurement

NIR fluorescence spectra of SWCNT-ssDNA were primarily acquired from 900 to 1600 nm with a NS MiniTracer spectrophotometer (Applied NanoFluorescence; Houston, TX). The 50 mW laser source had an excitation wavelength of 638 nm. Discrete NIR fluorescence spectra were also acquired with a custom ClaIR plate reader (Photon etc; Montreal, QC) with laser source excitation wavelengths of 655 and 730 nm (power of ∼1750 mW). Continuous fluorescence spectra were acquired with a custom IRina probe (Photon Etc; Montreal, QC). The laser source had an excitation wavelength of 655 nm. From each NIR spectrum acquired, SWCNT (7,5), (7,6), and (9,5) chiralities were analyzed with a custom MATLAB code which fit each emission peak with a Voigt model to obtain center wavelength and maximum intensity values.^10,29,43^*R*^2^ values for all fits were at least 0.98.

### Evaluation of SWCNT-Aptamer Sensitivity to IL-6

SWCNT-ssDNA of four sequences was initially screened for sensitivity to IL-6:15Apt, 31Apt, (GT)_15_, and (GT)_15_+31Apt. SWCNT-ssDNA were tested in both buffer conditions (0.5 mg/L SWCNT in 1× PBS) and serum conditions (0.5 mg/L SWCNT in 1× PBS + 10% FBS). Baseline fluorescence measurements were taken with the MiniTracer as described above. After baseline measurements, 5250 ng/mL human recombinant IL-6 protein (Invitrogen; Waltham, MA) was added to the experimental group and an equal volume of 1× PBS was added to the control group. All samples had a total volume of 120 μL and were performed in triplicate. Fluorescence spectra were acquired with the MiniTracer at 2, 15, 30, 60, 90, 120, 150, and 180 min after antigen addition. Data were processed in MATLAB as previously described.

### Evaluation of Sensor Sensitivity and Dynamic Range

Due to maximal sensitivity with the 31Apt sequence, further testing was performed with this construct. 0.5 mg/L SWCNT-31Apt was tested against various concentrations of IL-6 protein in buffer conditions in triplicate: 2.1, 21, 105, 210, 2100, 5250, and 8400 ng/mL. Fluorescence spectra were acquired with the ClaIR plate reader every 15 min for 3 h following antigen addition.

### Evaluation of Sensor Specificity

In addition to IL-6, the nanosensor was also tested against 5250 ng/mL of IL-1β and 5250 ng/mL of TNF-α in buffer conditions in triplicate. Fluorescence spectra were acquired with a MiniTracer as previously described.

### Kinetic Response

To evaluate the kinetics of the SWCNT-31Apt response to IL-6, continuous fluorescence spectra were acquired with the IRina probe. Baseline spectra were continually acquired for 300 s, 5250 ng/mL IL-6 was added to 0.5 mg/L SWCNT-31Apt, and then spectra were continually acquired for an additional 900 s.

#### Studies to Understand the SWCNT-Aptamer Sensing Mechanism

##### Thermal Denaturation and Refolding of DNA Aptamer Construct

SWCNT-31Apt constructs were heated to 95 °C in a Thermomixer F1.5 (Eppendorf; Hamburg, Germany) for 5 min and then cooled on the bench until they reached room temperature (∼15 min) to induce reversible thermal denaturation and refolding of the aptamer. 0.5 mg/L of the heat-treated SWCNT-31Apt constructs were tested against 5250 ng/mL IL-6 in buffer conditions, and fluorescence spectra were acquired in triplicate with the MiniTracer as previously described.

##### Surface Passivation with BSA

Prior work has shown that surface passivation of SWCNT with BSA can decrease nonspecific interactions of nanosensors in serum via adsorption to exposed regions of the nanotube.^42,43^ SWCNT-31Apt was incubated with BSA for 30 min at a 1:50 mass ratio on ice. The passivated nanosensors were then tested in triplicate against 5250 ng/mL of IL-6 under serum conditions. Fluorescence spectra were acquired with the MiniTracer as previously described.

##### Noncovalent Functionalization with SDBS

Prior work has shown SDBS-mediated enhancement of SWCNT-ssDNA sensor response via surface adsorption to exposed regions of the nanotube surface.^10^ 0.5 mg/L SWCNT-31Apt was tested against 5250 ng/mL IL-6 in 1× PBS with the addition of 0.2% SDBS. Fluorescence spectra were acquired in triplicate with the MiniTracer as previously described.

### Baseline Sensor Stability

To assess the baseline stability and shelf life of the nanosensors, a batch of SWCNT-31Apt was prepared as above, excluding the step of filtration, and stored at 4 °C for 14 days. On days 1, 2, 3, 7, 8, 9, 12, 13, and 14 following suspension preparation, a 30 μL aliquot was removed from the stock solution, filtered with a 100 kDa Amicon centrifugal filter as outlined above, and diluted to 0.5 mg/L in 1× PBS. Immediately following dilution, fluorescence spectra of SWCNT-31Apt were acquired in triplicate every 15 min for 3 h using the ClaIR plate reader.

### DNA Displacement Study

To further understand the mechanism of the SWCNT-31Apt sensor and evaluate the stability of the SWCNT/aptamer/antigen complexes, a DNA displacement study was performed using cyanine dye-functionalized ssDNA. SWCNT-ssDNA suspensions were prepared as previously described using (GT)_15_+Cy3 or 31Apt+Cy5. Each SWCNT-ssDNA construct was incubated overnight at 4 °C under one of three conditions: 1× PBS, 1× PBS + 5250 ng/mL IL-6 protein, or 1× PBS + 2.5% DOC, which is a positive control for displacing DNA from SWCNT. After 18 h of incubation, all samples were subjected to centrifugal filtration with a 100 kDa Amicon centrifugal filter. The SWCNTs remaining inside the filter were discarded, and the flow-through was collected for further analysis. The visible absorbance of all solutions was measured with a Biotek microplate reader with Gen5 software (BioTek; Winooski, VT). Data were analyzed in Microsoft Excel.

#### Investigation of Sensor Functionality in an Activated Macrophage Disease Model

The RAW 264.7 murine macrophage cell line (American Type Culture Collection; Gaithersburg, MD) was seeded in 6-well plates at 100,000 cells/well. Cells were cultured with DMEM supplemented with 10% FBS and 1% penicillin-streptomycin until they reached 80% confluency. Each well was treated with 2.5 μg/mL LPS isolated from*E. coli* 0111:B4 (MilliporeSigma; Burlington, MA) in a 1.3 mL total volume. Cells were then incubated for 24 h to induce macrophage activation and cytokine release.^49^ ELISA (Invitrogen, Waltham, MA, Product #88-7066-22) was performed to quantify the level of IL-6 present in the cell media. Conditioned media were collected and frozen at −20 °C until use. 0.5 mg/L SWCNT-31Apt was added to thawed conditioned media samples from 0 and 2.5 μg/mL LPS treatment groups as well as fresh DMEM + FBS as a control. Samples were evaluated in triplicate, and fluorescence spectra were acquired with the MiniTracer as described above.

### Data Analysis

NIR fluorescence corresponding to individual nanotube chirality emission peaks was fit with a custom MATLAB code to a Voigt model to determine their center wavelength and maximum intensity values (MATLAB code is available upon request). Changes in the SWCNT center wavelength and maximum intensity values were reported relative to their emission prior to antigen addition. Means for triplicate samples plus the standard deviation were obtained. Statistical significance was determined with a two-sample *t* test with a Welch correction.

## Results and Discussion

### Screening of the Optimal Aptamer Sensor Format

**Figure 1:**
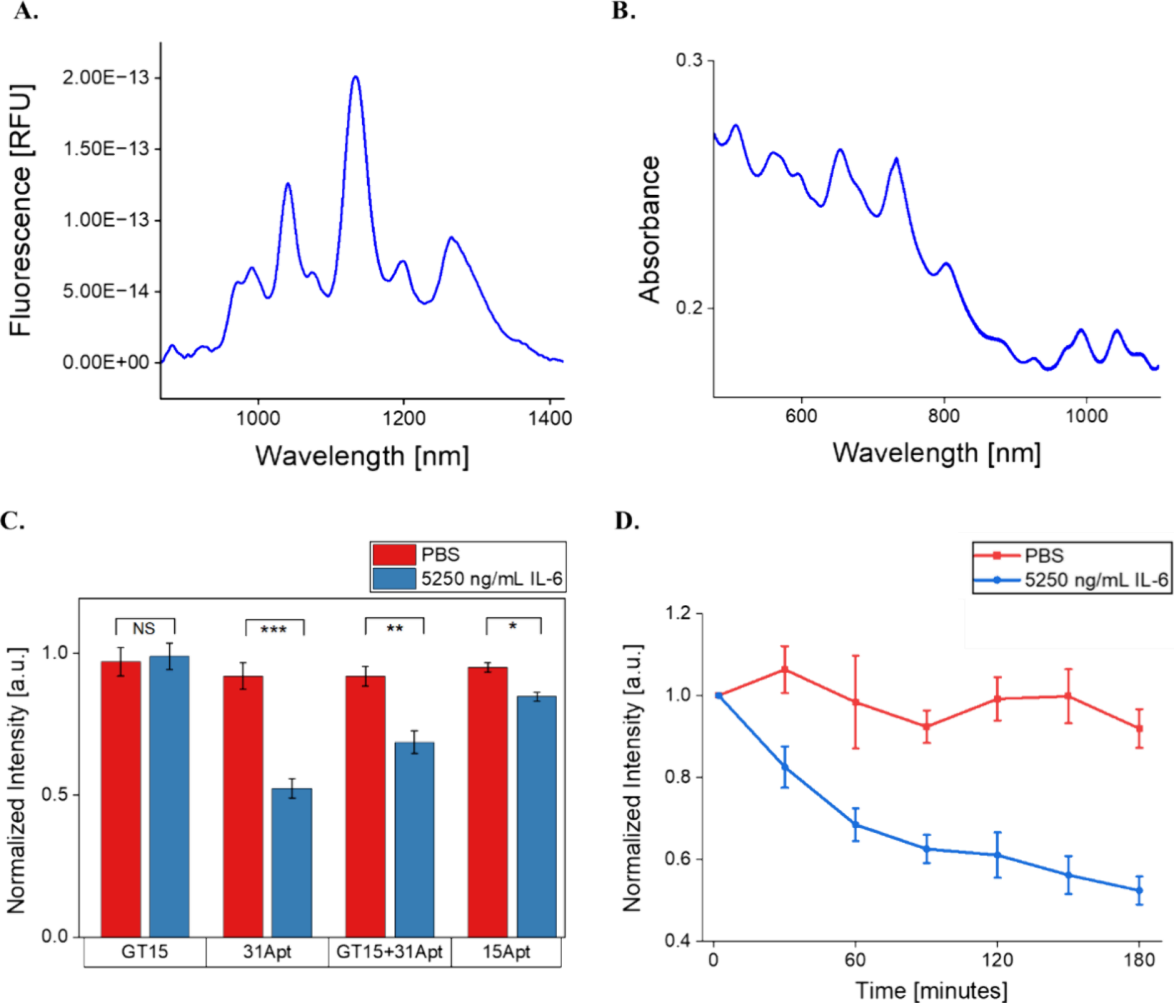
Evaluation of sensor synthesis and response to IL-6 in PBS. (A) Representative NIR fluorescence spectrum of the SWCNT-31Apt construct. (B) Representative Vis-IR absorbance spectrum of the SWCNT-31Apt construct. (C) (7,5) Fluorescence intensities of SWCNT-ssDNA in PBS after 3 h of exposure to IL-6. (GT)_15_: *p* = 0.66, 31Apt: *p* = 0.00055, (GT)_15_+31Apt: *p* = 0.0017, 15Apt: *p* = 0.0029. (D) (7,5) Fluorescence intensity of SWCNT-31Apt in response to IL-6 over 3 h (*n* = 3; all points represent mean ± standard deviation).

SWCNTs separately encapsulated with four ssDNA sequences were prepared and tested against 5250 ng/mL IL-6 in buffer conditions: (GT)_15_, (GT)_15_+31Apt, 31Apt, and 15Apt (Table 1). Optical characterization of nanosensor constructs showed effective suspension of SWCNT with ssDNA with bright, stable NIR fluorescence emission and excitation peaks (Figure 1A,B). In prior work, evaluation of the fluorescence response of 0.5 mg/mL nanosensor to 5250 ng/mL IL-6 was suitable to confirm nanosensor responsiveness.^29,50^ Analysis of the (7,5) SWCNT chirality showed that all control groups (containing only SWCNT-ssDNA in 1× PBS) retained a relatively stable fluorescence intensity over three h (100% normalized fluorescence). As expected, (GT)_15_ showed no response to IL-6 protein (*p* = 0.66), as it was used as a control sequence that binds to SWCNT well but with no known affinity for IL-6 (Figure 1C). The truncated 15Apt sequence demonstrated minimal response, quenching 15% (*p* = 0.0029). The fluorescence of (GT)_15_+31Apt quenched 32% (*p* = 0.0017), while 31Apt showed the most significant response, quenching 48% over three h (*p* = 0.00055). SWCNT-31Apt exhibited time-dependent quenching over 3 h, with the majority of the response occurring in the first hour (Figure 1D). Fluorescence from two additional SWCNT chiral species was analyzed—(7,6) and (9,5)—demonstrating nearly identical patterns of response: around 50% quenching over 3 h with no significant wavelength shifts (Figure S1). We also observed that the other three SWCNT-ssDNA constructs demonstrated similar time dependence relative to their total response (Figure S2).

Dispersion of the SWCNT with various proteins, synthetic polymers, surfactants, or oligonucleotides is an excellent way to solubilize the nanotubes and potentially impart increased biocompatibility and molecular sensitivity. However, this technique typically creates a trade-off between molecular specificity and quantum yield of the resulting nanosensors. SWCNTs dispersed in surfactants exhibit high quantum yield but have no inherent selectivity toward any particular analyte.^24,51^ Protein-encapsulated SWCNT has demonstrated successful differentiation between molecularly similar targets. However, protein encapsulation is limited by low dispersion efficiency and lack of concise control during protein immobilization, which can lead to unfavorable conformations for antigen binding.^52,53^ DNA is among the most well-studied wrappings for SWCNT sensors due to its ability for high dispersion efficiencies and customization of properties with various oligonucleotide sequences and structures. However, the majority of DNA-dispersed SWCNT formulations have random ssDNA sequence structures (such as GT repeats, as used here) with no specificity toward any particular analyte. DNA aptamers hold a unique advantage in this context: as oligonucleotides, they enable high-yield SWCNT dispersions; however, they can also provide high specificity toward a particular target.

31Apt was previously selected for its affinity for IL-6 via SELEX.^38^ The authors of that original study also evaluated a rationally designed truncation of that, which we termed 15Apt. However, the original evaluation of the full-length and truncated aptamers demonstrated that the IL-6 binding equilibrium constant of 31Apt was a full order of magnitude stronger than 15Apt (17 vs 190 nM, respectively). The full-length 31Apt has been previously validated for use in electrochemical and field-effect transistor sensors, while 15Apt has not.^50,54−56^ Indeed, primary folding structures of 15Apt are predicted to be less stable than those of 31Apt (UNAFold server^57^). (GT)_15_ is known to stably disperse SWCNT but does not have demonstrated affinity for IL-6.^58^ Therefore, a comparison of IL-6 responsiveness for the full-length aptamer with that of the truncated version and the negative control met expectations for this study.

The results from the (GT)_15_+31Apt construct, however, were interesting to note. Previous work has shown the enhancement of SWCNT-DNA sensors through the use of heterobifunctional DNA with two domains: an anchor sequence, which adheres to the SWCNT surface, and a molecular recognition sequence, which binds and captures the target protein.^39^ We explored this strategy with (GT)_15_ serving as an anchor sequence and 31Apt as the recognition sequence. However, the (GT)_15_+31Apt sensors exhibited a somewhat less robust response, though still significant, compared to that of 31Apt alone. This implies that noncovalent adsorption of 31Apt to the nanotube surface does not inhibit the accessibility of IL-6 to its active site and further suggests that interaction of the aptamer with the SWCNT surface is itself important in the mechanism of sensor function. We hypothesize that this may be due to two possible reasons. The first is that the addition of the (GT)_15_ anchor domain alters the tertiary structure of the aptamer, which may be possible as all prior studies with this aptamer only used the full-length 31Apt.^50,54−56^ Indeed, the folding structure of 31Apt is slightly more stable than that of the (GT)_15_-31Apt sequence, wherein the addition of (GT)_15_ is not predicted to contribute to the tertiary structure but rather create a long tail (UNAfold server).^57^ The second possible explanation is that the anchor domain may inhibit the rearrangement of the aptamer sequence on the SWCNT surface upon IL-6 binding. This is theoretically possible as the freedom to bind and rearrange upon binding to its cognate analyte is an important feature in many aptamer-based sensors.^59,60^ 31Apt alone was used for subsequent experiments.

**Figure 2:**
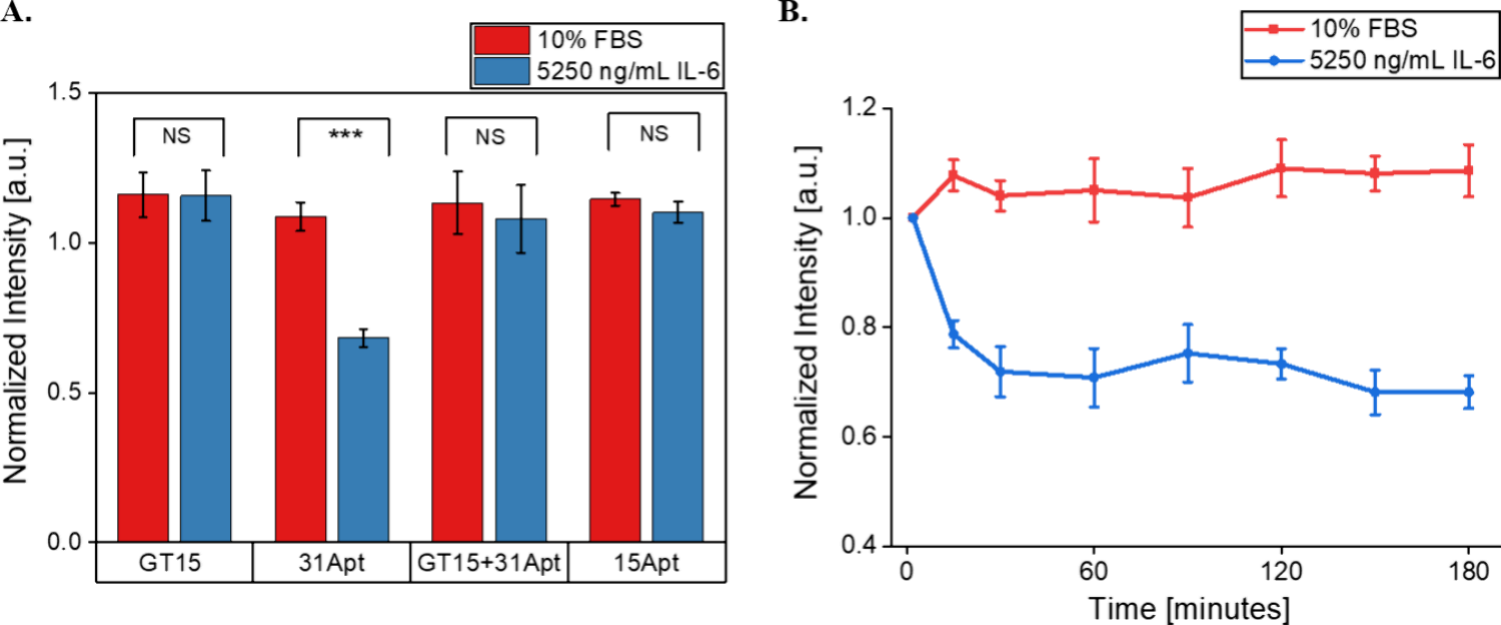
Sensor response to IL-6 in PBS + 10% FBS. (A) (7,5) chirality fluorescence intensities of SWCNT-ssDNA after 3 h of exposure to IL-6 + 10% FBS. (GT)_15_: *p* = 0.95, 31Apt: *p* = 0.00062, (GT)_15_+31Apt: *p* = 0.58, 15Apt: *p* = 0.15. (B) (7,5) chirality fluorescence intensity of SWCNT-31Apt over 3 h in response to 5250 ng/mL IL-6 + 10% FBS (*n* = 3; all points represented mean ± standard deviation).

The same four SWCNT-ssDNA constructs were tested under 10% serum to investigate their function in more complex protein environments. The fluorescence of the (7,5) SWCNT chirality dispersed with 31Apt quenched 32% within 3 hours in response to 5250 ng/mL IL-6 protein, slightly less than in buffer alone (*p* = 0.00062) (Figure 2A). Interestingly, none of the other ssDNA sequences tested demonstrated a significant response ((GT)_15_: *p* = 0.95, (GT)_15_+31Apt: *p* = 0.58, 15Apt: *p* = 0.15). Quenching of 31Apt reached its maximum within 30 min, after which it was stable (Figure 2B). No other SWCNT-ssDNA construct demonstrated any substantial differences across 3 hours (Figure S3). We anticipated this finding as protein corona formation is known to hinder sensor response.^29^ Therefore, the SWCNT constructs that demonstrated lower responsiveness in buffer were no longer functional in serum. It is notable that the 31Apt-SWCNT construct still demonstrated a substantial and significant response, indicating that it formed a stable complex with IL-6 on the SWCNT surface, even in the presence of a complex protein corona. It is clear that this was not the case for either the 15Apt or (GT)_15_-Apt31 constructs, likely due to the above-described reduced stability of both DNA constructs.

### Sensitivity and Dynamic Range of the SWCNT-31Apt Sensor

**Figure 3:**
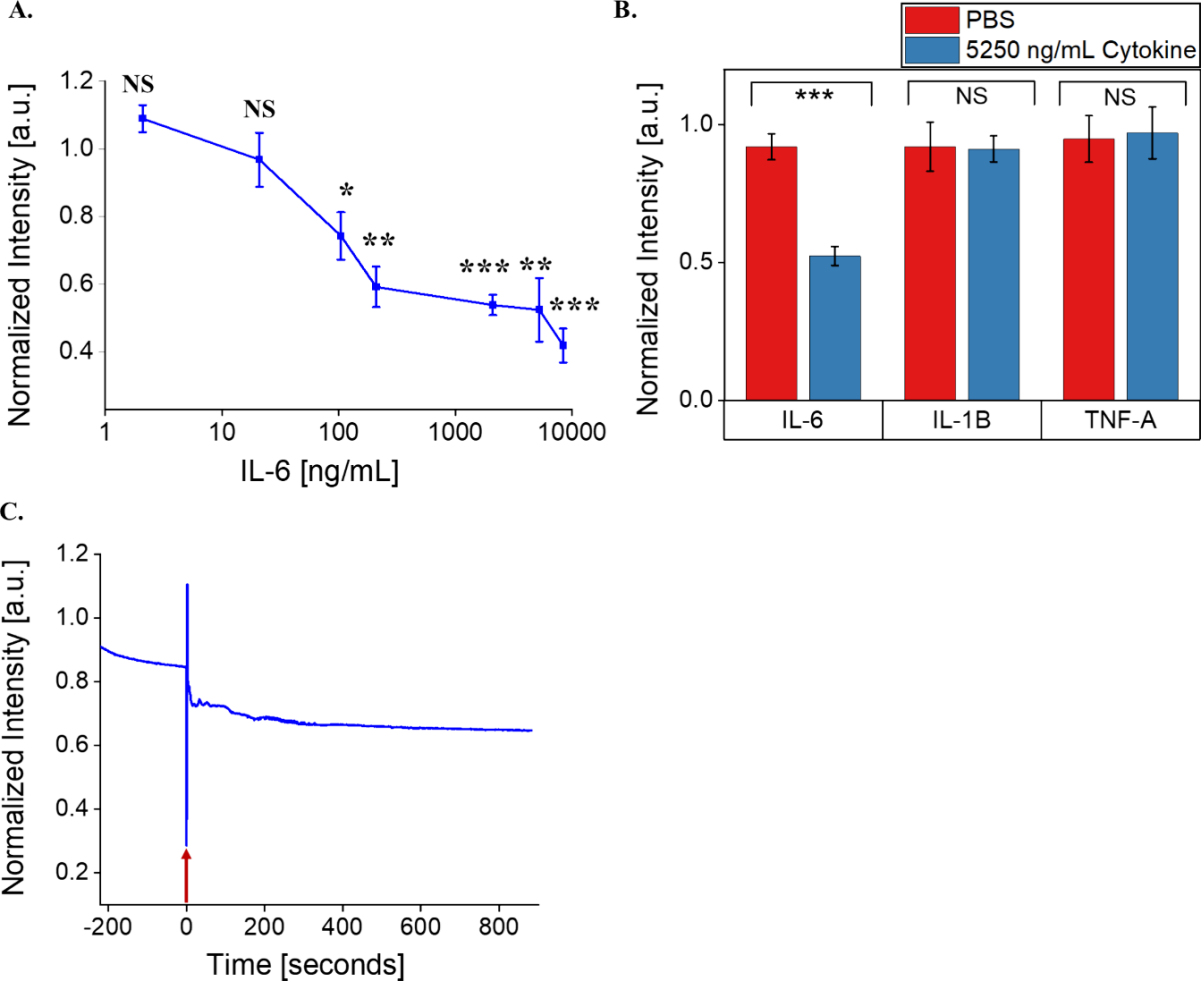
Determination of nanosensor sensitivity, selectivity, and rapidity. (A) (7,5) Fluorescence response of SWCNT-31Apt in PBS as a function of IL-6 concentration. (B) (7,5) Fluorescence intensity of SWCNT-31Apt in PBS after 3 h of exposure to 5250 ng/mL IL-6, IL-1β, and TNF-α (*n* = 3; all points represent mean ± standard deviation). (C) Fluorescence intensity of SWCNT-31Apt in PBS immediately before and after the addition of 5250 ng/mL IL-6. Moment of protein addition (time 0) indicated by the red arrow.

Given its robust response in serum, we next evaluated the sensitivity of the SWCNT-31Apt sensor to respond to a range of IL-6 concentrations in PBS. At or below 21 ng/mL IL-6, SWCNT-31Apt did not show any significant quenching of photoluminescence (2.1 ng/mL: *p* = 0.067, 21 ng/mL: *p* = 0.42) (Figure 3A). At or above 105 ng/mL IL-6, SWCNT-31Apt demonstrated quenching of fluorescence intensity that was monotonic to the sample concentration of IL-6 (105 ng/mL: *p* = 0.028, 210 ng/mL: *p* = 0.0022, 2100 ng/mL: *p* = 0.00071, 5250 ng/mL: *p* = 0.0078, and 8400 ng/mL: *p* = 0.00023). The highest concentration tested, 8400 ng/mL, induced the most dramatic response (58% fluorescence quenching). We therefore conclude that this sensor construct is quantitative. Pathological levels of IL-6 in biofluids span 0.005–500 ng/mL, depending on the fluid and disease.^61^ The nanosensor demonstrated a monotonic response between 105 and 8400 ng/mL, with a limit of detection within the clinically relevant range.

### Specificity of the SWCNT-31Apt Sensor

In order to determine the selectivity of the SWCNT-31Apt construct for IL-6 and not other inflammatory cytokines in an inflammatory disease context, we tested its response against two other cytokines. TNF-α and IL-1β were chosen as they are prominent representatives of two separate cytokine structural families compared to IL-6, which are typically found in similar biological contexts.^62^ All three classical inflammatory cytokines play important roles in both acute and chronic inflammatory processes.^4^ TNF-α, IL-1β, and IL-6 can have synergistic effects on inflammation, influence signaling pathways through cross-talk, and are known therapeutic targets for inflammatory disease.^2,63^ In light of this, we found that the SWCNT-31Apt nanosensor demonstrated sensitivity toward IL-6 but did not respond to TNF-α or IL-1β (Figure 3B). This demonstrates the selectivity of the nanosensor and highlights its ability to distinguish IL-6 from other inflammatory cytokine families.

### Rapid Kinetic Response

Continuous acquisitions of fluorescence spectra of SWCNT-31Apt showed that the quenching of photoluminescence begins within seconds of IL-6 addition (Figure 3C). 25% fluorescence quenching occurred within 15 s of antigen addition. Fluorescence intensity steadily decreased: 30% quenching within 115 s and 35% quenching within 560 s of antigen addition. This finding is consistent with the previous time-dependent response of the sensor (Figures 1D and 2B), where at least 20% quenching occurs within 15 min of antigen addition and quenching continues steadily over the course of three hours. Detection and quantification of biomarkers with temporal resolution are beneficial for clinical and research applications. Due to its ability to respond rapidly to IL-6, this nanosensor construct could be used in immunology research to study the kinetics associated with cytokine release in inflammatory disease states. Similarly, dynamic and time-sensitive measurements of biomarkers are ideal for clinical applications that require continuous monitoring.

### Investigation of the Sensing Mechanism

We performed several modifications to the standard assays above to further understand the mechanism by which the SWCNT-aptamer complex responds with high sensitivity, specificity, and speed to IL-6. Typically, in DNA aptamer selection experiments, the library is heated to 95 °C and cooled back to room temperature to ensure a proper tertiary structure is adopted. We therefore tested whether denaturation and folding affected the sensor function. However, after heating to 95 °C and cooling back to room temperature, the SWCNT-31Apt construct no longer exhibited the same response to IL-6. Rather, it showed only 10% quenching after 3 h (*p* = 0.032) (Figure S4A). This suggests that the probe-tip sonication of aptamer with SWCNT is sufficient for proper folding and that additional perturbation of this interaction disrupts the ability to respond to IL-6.

Surface adsorption of bovine serum albumin (BSA) has been shown to enhance the functionality of an antibody-based SWCNT sensor through adsorption to exposed surfaces of the nanotube.^42,43,64^ We therefore evaluated whether such adsorption to an exposed SWCNT surface would perturb the sensor response to IL-6. In this study, BSA passivation did not enhance the selectivity of SWCNT-31Apt when deployed in serum conditions (Figure S4B). In fact, there was no significant difference in the fluorescence intensity of BSA-incubated SWCNT-31Apt with and without the presence of IL-6 in serum conditions (*p* = 0.45). We therefore conclude that this study supports our hypothesis that the SWCNT interaction with the 31Apt sequence is essential to IL-6 sensing, as perturbation of that interaction with a large BSA molecule (66 kDa compared to ∼19 kDa of the aptamer) interferes with such a response.

Similarly, additional prior work has investigated the interaction of surfactants with the nanotube surface.^51^ As an example, a previous SWCNT sensor for microRNA was substantially enhanced due to the interaction of the hydrophobic surfactant sodium dodecylbenzene sulfate (SDBS) with exposed SWCNT surface and replacing ssDNA removed from the SWCNT surface.^10^ In this study, however, SWCNT-31Apt showed no significant response to IL-6 in the presence of 0.02% SDBS (*p* = 0.087) (Figure S4C). We believe that this result further supports our hypothesis that 31Apt is not removed from the SWCNT surface and that the 31Apt sequence must be free to interact or potentially rearrange on the nanotube surface to exhibit detection of IL-6.

### Stability of the Sensor

**Figure 4:**
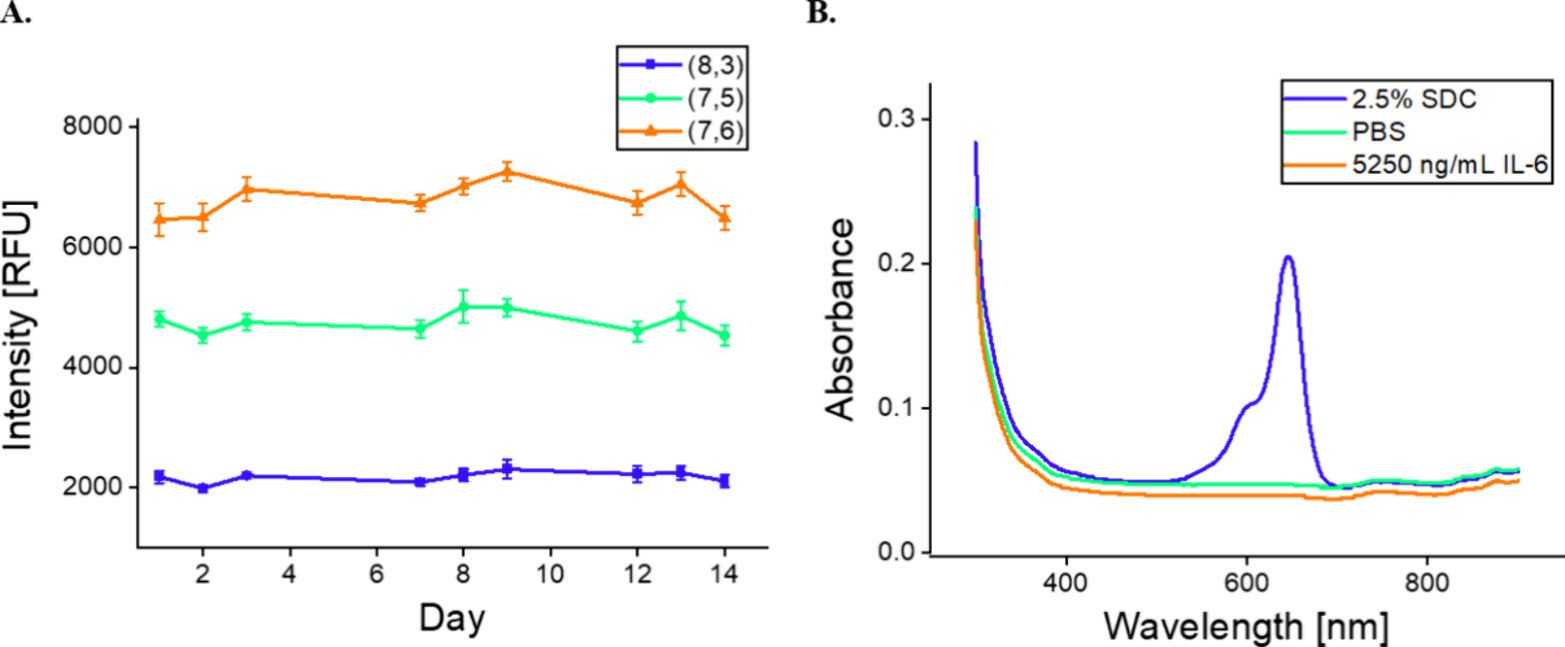
Stability of nanosensor constructs. (A) (8,3), (7,5), and (7,6). Fluorescence intensity of SWCNT-31Apt in PBS for 2 weeks after preparation (*n* = 3; all points represent mean ± standard deviation). (B) Absorbance of flow-through from SWCNT-31Apt+Cy5 after overnight incubation.

To further investigate the mechanism of sensor function and to understand its stability, we first investigated its stability over the course of 14 days. To do so, we measured the baseline fluorescence of SWCNT-31Apt, which found considerable stability. Fluctuations of all chiralities analyzed—the (8,3), (7,5), and (7,6)—were minimal and within 10% variation (Figure 4A). This suggests that the SWCNT-31Apt construct is stable for at least 2 weeks when stored at 4 °C.

We next studied whether the interaction of 31Apt with IL-6 displaces the aptamer sequence from the surface of the nanotube. One possibility for the mechanism of sensing was that the sensor quenching response may be due to such a displacement and subsequent SWCNT aggregation. In this experiment, the results suggested that IL-6 protein does not displace 31Apt from the SWCNT surface during the sensor response. When SWCNT functionalized with cyanine, dye-labeled ssDNA ((GT)_15_+Cy3 or 31Apt+Cy5) was incubated overnight with 2.5% of the surfactant deoxycholate (DOC) as a positive control, DNA was displaced from the nanotube surface as evidenced by fluorescence flow-through of the centrifugal filter membrane (Figure S5). The same nanotube constructs incubated with PBS as the negative control demonstrated no DNA displacement. SWCNT-31Apt+Cy5 saw no measurable DNA displacement after overnight incubation with 5250 ng/mL IL-6 protein (Figure 4B). This is promising evidence for future applications of the sensor, where long-term storage and stable binding would be beneficial as well as the potential for sensor reversibility. Importantly, this result demonstrates that the interaction of IL-6 with the aptamer occurs on the surface of the nanotube and is not independent of it. This further suggests that 31Apt may undergo rearrangement on the SWCNT surface to allow for IL-6 binding and fluorescence response but does not undergo dissociation or release.

### Investigation of Sensor Functionality in Activated Macrophages

**Figure 5:**
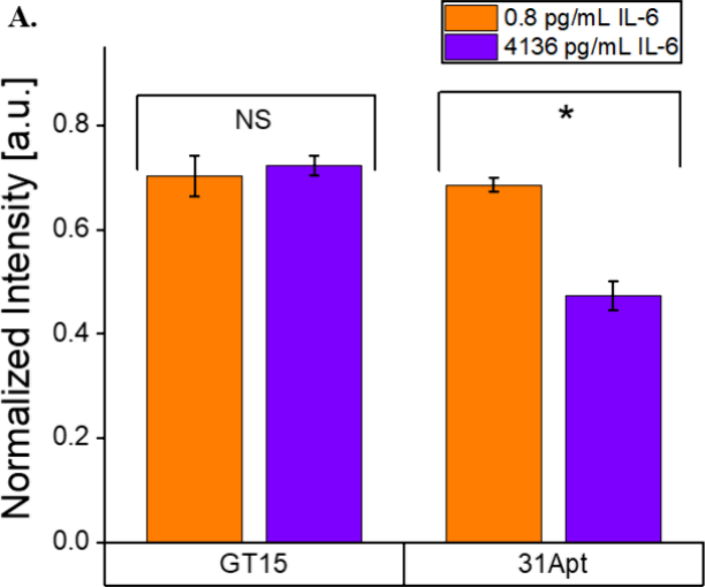
Quantification of IL-6 in conditioned media from LPS-activated macrophages. (7,5) fluorescence intensity of SWCNT-(GT)_15_ and SWCNT-31Apt after 3 h of exposure to conditioned media from Raw 264.7 cells. SWCNT-31Apt distinguished activated and nonactivated macrophages (*p* = 0.0014), while SWCNT-(GT)_15_ did not (*p* = 0.48) (*n* = 3; all points represent mean ± standard deviation).

To evaluate the function of the SWCNT-31Apt nanosensor construct using an in vitro model of disease, in this case, acute bacterial infection, we exposed the sensor to conditioned cell media. The sensor was exposed to nonconditioned media as a baseline control, media recovered with naïve RAW 264.7 murine macrophages, or media recovered from the same macrophages activated with bacterial lipopolysaccharides (LPS). By ELISA, we found that macrophages treated with 2.5 μg/mL LPS for 24 h expressed 4135.9 pg/mL IL-6 in their conditioned media, while macrophages not treated with LPS expressed 0.8 pg/mL IL-6 in their conditioned media. The nanosensor demonstrated a 22% decrease in fluorescence from the non-LPS conditioned media to the LPS conditioned media (*p* = 0.0014) (Figure 5). The observed response aligns with our previously constructed concentration–response curve, as 20% quenching would be expected at the given concentration of IL-6 (Figure 3A). As previously observed, the majority of the sensor response was observed in the first 15 min and then continued steadily over 3 h (Figure S6A).

To determine the role of 31Apt in the observed sensor response, we also performed an experiment with SWCNT-(GT)_15_. This nonsensing control exhibited no significant difference in the non-LPS conditioned media and LPS conditioned media (*p* = 0.48) (Figures 5 and S6B), demonstrating the importance of the IL-6-specific aptamer in sensor functionality. As we previously found that this nanosensor is highly specific to IL-6, having demonstrated a consistent response in 10% FBS and no response to other cytokines, we conclude that this response is IL-6 specific in conditioned media despite the presence of other cytokines.^49^ We were also excited to find that the sensor responds as well to full-length mouse IL-6 as it did to recombinant human IL-6 in the above studies.

## Conclusions

In this work, we designed and investigated the use of a novel IL-6 aptamer-based sensor complexed with SWCNT as an optical transducer. Sensor construction was straightforward with no intermediary steps, and we found that the sensor was most optimally functional with no hybridization sequence or truncation. We also found that the sensor response requires the IL-6 aptamer and not a random ssDNA sequence. We further investigated the sensitivity, selectivity, rapidity, and mechanism of action/stability of the nanosensor. The sensor demonstrated a clinically relevant detection limit of 105 ng/mL and responded specifically to IL-6 in buffer and serum conditions.^61^ This nanosensor construct exhibited a rapid response to its target and formed a stable construct with IL-6 without displacing the aptamer from the SWCNT surface. Excitingly, we demonstrated that this nanosensor can detect bacterial infection, as modeled by LPS-incubated macrophages in vitro.

Ongoing work is likely to push this novel sensor further toward utility both in the clinic and as a research tool. It may be possible to develop multiplexed nanosensors that detect several cytokines with chirally separated SWCNT, which may also improve sensitivity and specificity of the sensor.^65,66^ It may also find utility as in prior work in SWCNT-based sensing of extracellular analytes via sensor immobilization on a glass substrate upon which cells are cultured.^67^ Further in vivo development may be possible through embedding the sensor within a semipermeable membrane or hydrogel.^43,68^ We anticipate that a combination of these approaches may confer clinical utility in early stage detection of chronic inflammatory diseases or the rapid detection of infection.

## Supporting Information Available

Time-dependent response of additional chiralities of the nanosensor and other constructs to IL-6 (PDF)
