# Supplementary material for: Optical Aptamer-Based Cytokine Nanosensor Detects Macrophage Activation by Bacterial Toxins
Source: ACS Sens. 2024 Jun 27;9(7):3697–706. doi: 10.1021/acssensors.4c00887 (PMC11287749; doi:10.1021/acssensors.4c00887)
Supplement: Supplementary file 1 — se4c00887_si_001.pdf [file se4c00887_si_001.pdf]

Supporting Information for:

An optical aptamer-based cytokine nanosensor detects macrophage activation by bacterial toxins

Amelia K. Ryan[1], Syeda Rahman[1], Ryan M. Williams[1,2]*

[1]The City College of New York, Department of Biomedical Engineering, New York, NY 10031
[2]PhD Program in Chemistry, Graduate Center, City University of New York, New York, NY 10016
*correspondence to rwilliams4@ccny.cuny.edu

Supporting Figures

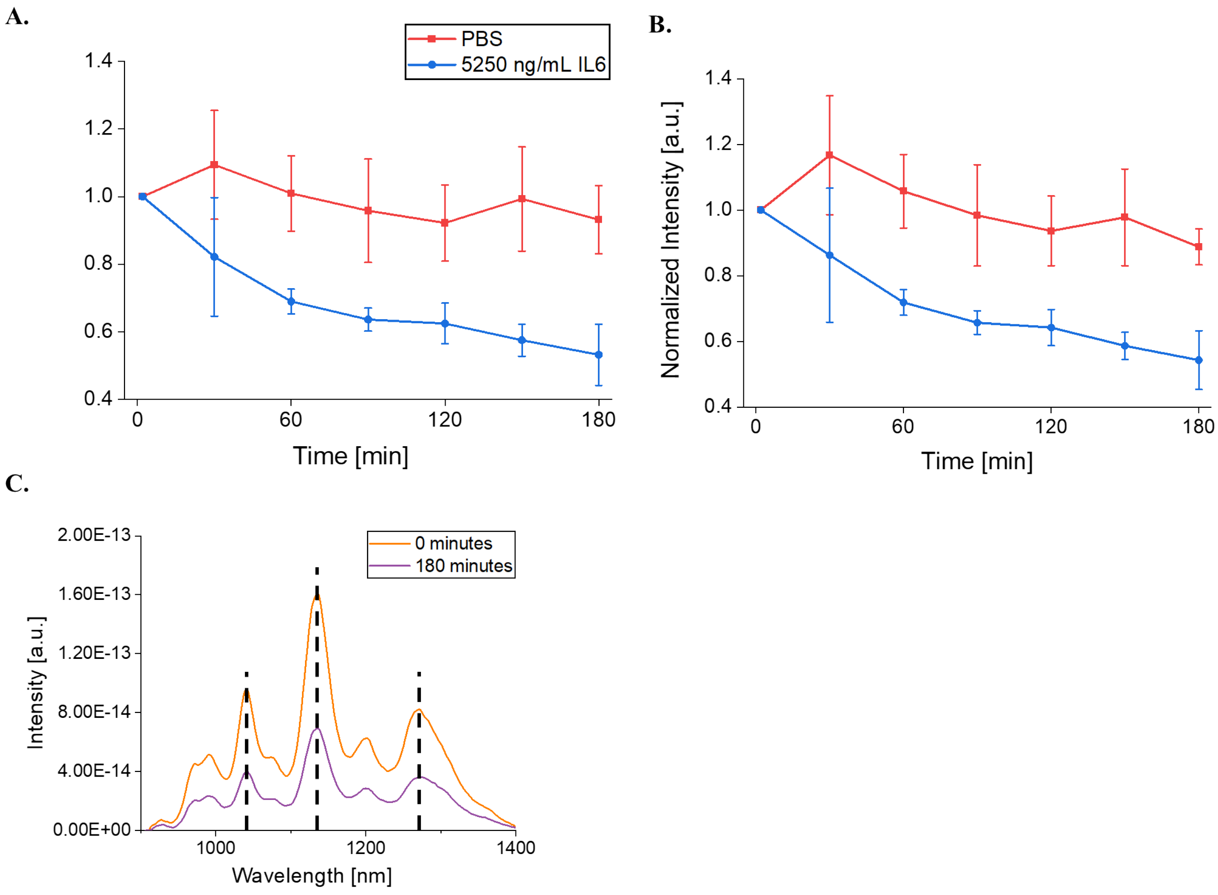

Figure S1. Time-dependent response of each SWCNT-31Apt chirality to IL-6 in PBS. A.. (7,6) fluorescence intensity over three hours. B. (9,5) fluorescence intensity over three hours. C. Full NIR spectra of SWCNT-31Apt before the addition of IL-6 (0 minutes) and after three hours of incubation with IL-6 (180 minutes). Notably, the fluorescence intensity of all chiralities decreases after exposure to IL-6 but no significant wavelength shifts are observed (n = 3; mean ± standard deviation).

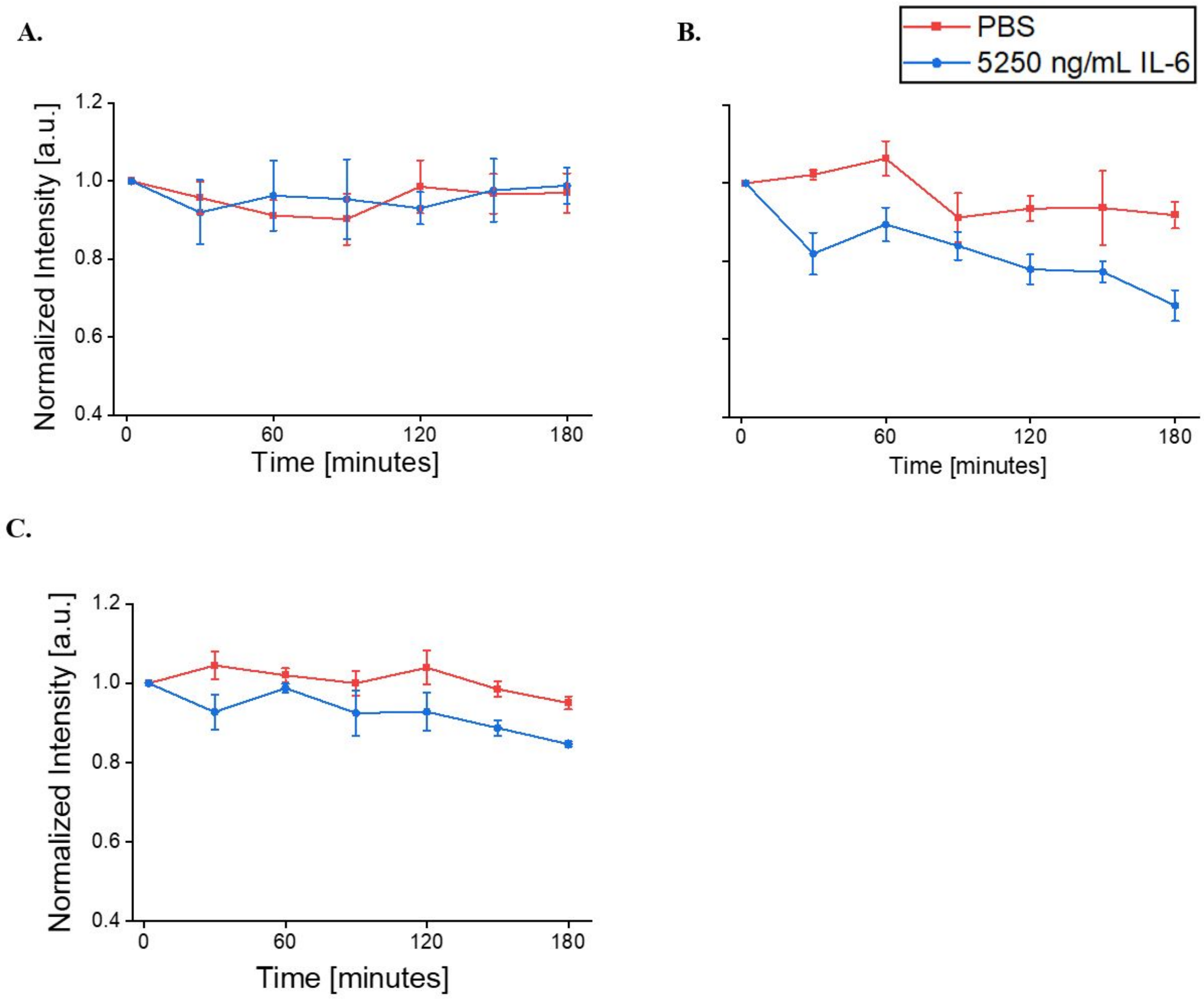

Figure S2. **Time-dependent response of SWCNT-ssDNA constructs to IL-6 in PBS.** A. (7,5) fluorescence intensity of SWCNT-$(GT)_{15}$ over three hours. B. (7,5) fluorescence intensity of SWCNT-$(GT)_{15}$+31Apt over three hours. C. (7,5) fluorescence intensity of SWCNT-15Apt over three hours (n = 3; mean ± standard deviation).

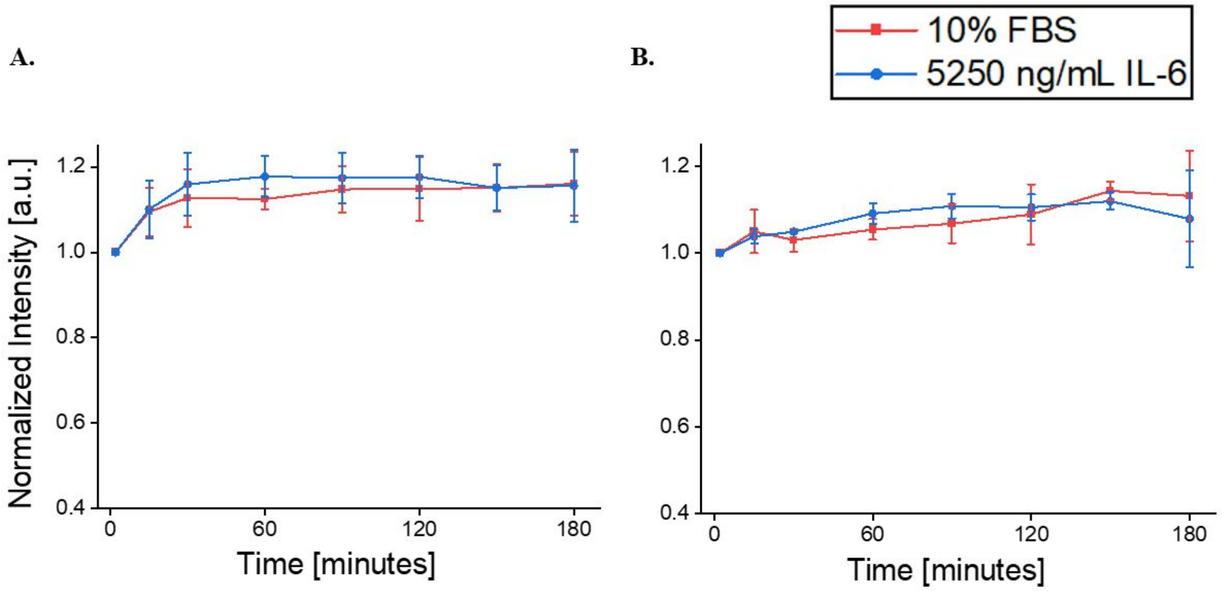

Figure S3. Time-dependent response of SWCNT-ssDNA constructs to IL-6 in PBS + 10% FBS. A. (7,5) fluorescence intensity of SWCNT-$(GT)_{15}$ over three hours. B. (7,5) fluorescence intensity of SWCNT-$(GT)_{15}$+31Apt over three hours. C. (7,5) fluorescence intensity of SWCNT-15Apt over three hours (n = 3; mean ± standard deviation).

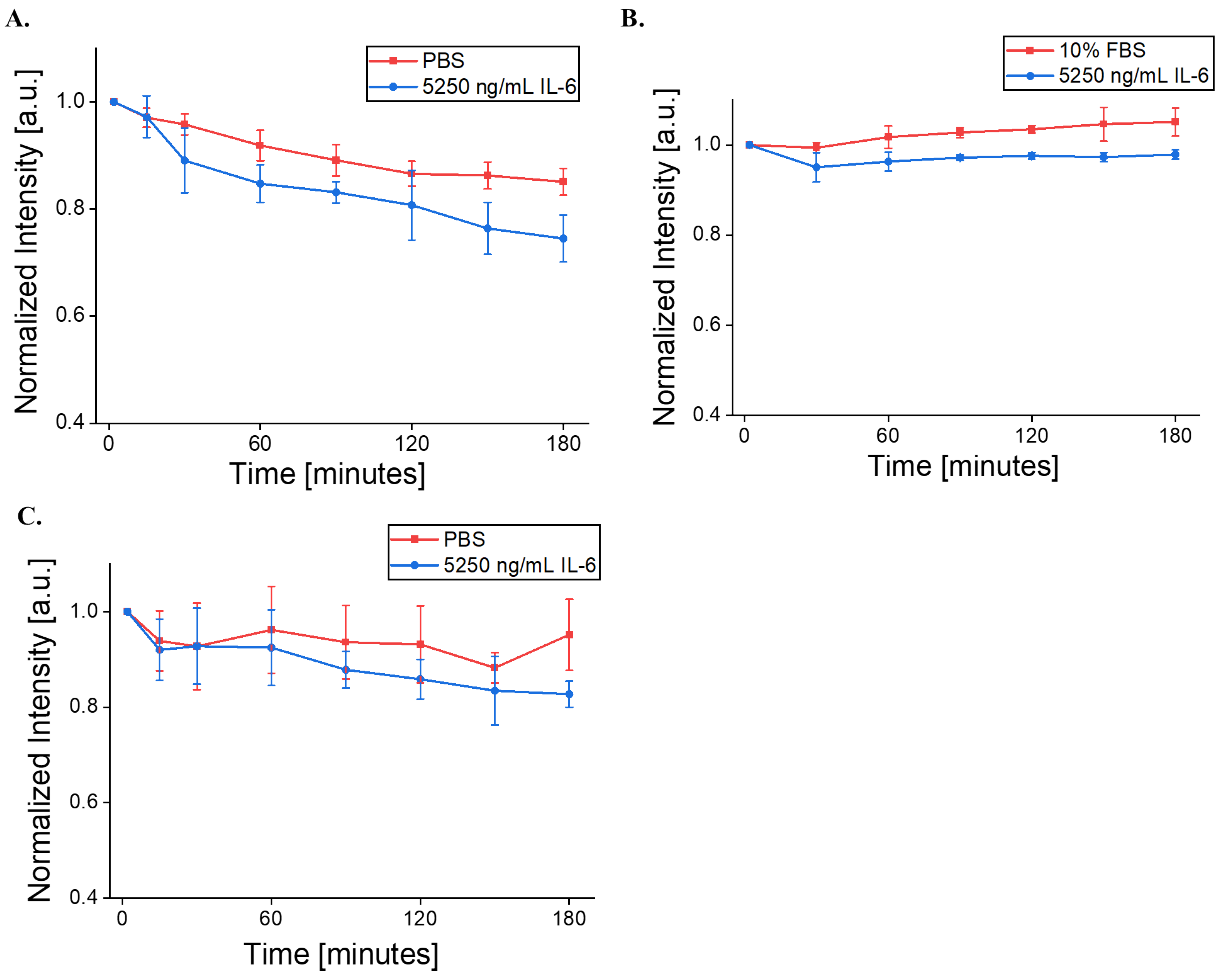

Figure S4. Mechanistic modulations of nanosensor. A. (7,5) Fluorescence intensity of heat-treated SWCNT-31Apt in response to IL-6 in PBS over three hours. B. (7,5) Fluorescence intensity of BSA-passivated SWCNT-31Apt in response to IL-6 in PBS + 10% FBS over three hours. C. (7,5) Fluorescence intensity of 0.02% SDBS-coated SWCNT-31Apt in response to IL-6 in PBS over three hours (n = 3; mean ± standard deviation).

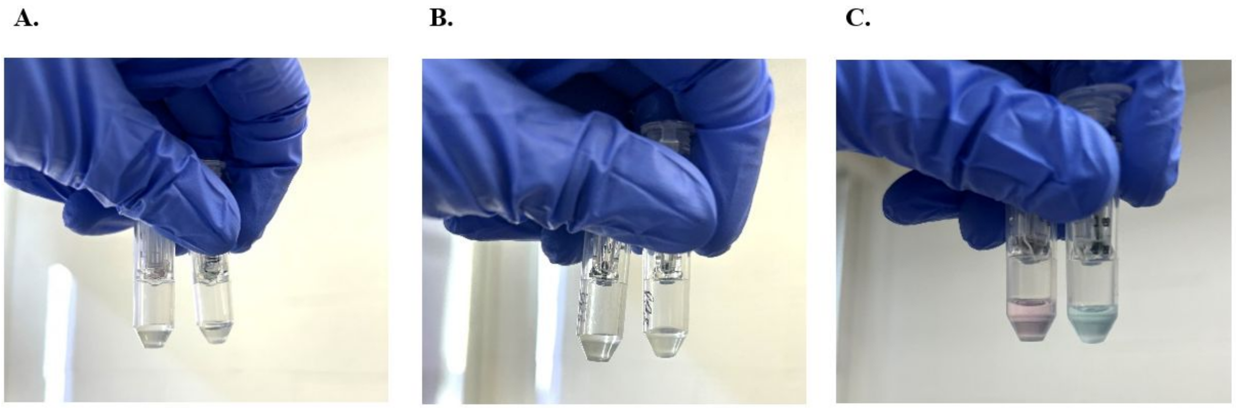

Figure S5. Photos of displaced cyanine dye-labeled DNA. A. Flow-through after incubation of SWCNT-$(GT)_{15}$+Cy3 (left) and SWCNT-31Apt+Cy5 (right) with PBS. B. Flow-through after incubation of SWCNT-$(GT)_{15}$+Cy3 (left) and SWCNT-31Apt+Cy5 (right) with 5250 ng/mL IL-6 protein in PBS. C. Flow-through after incubation of SWCNT-$(GT)_{15}$+Cy3 (left) and SWCNT-31Apt+Cy5 (right) with 2.5% DOC.

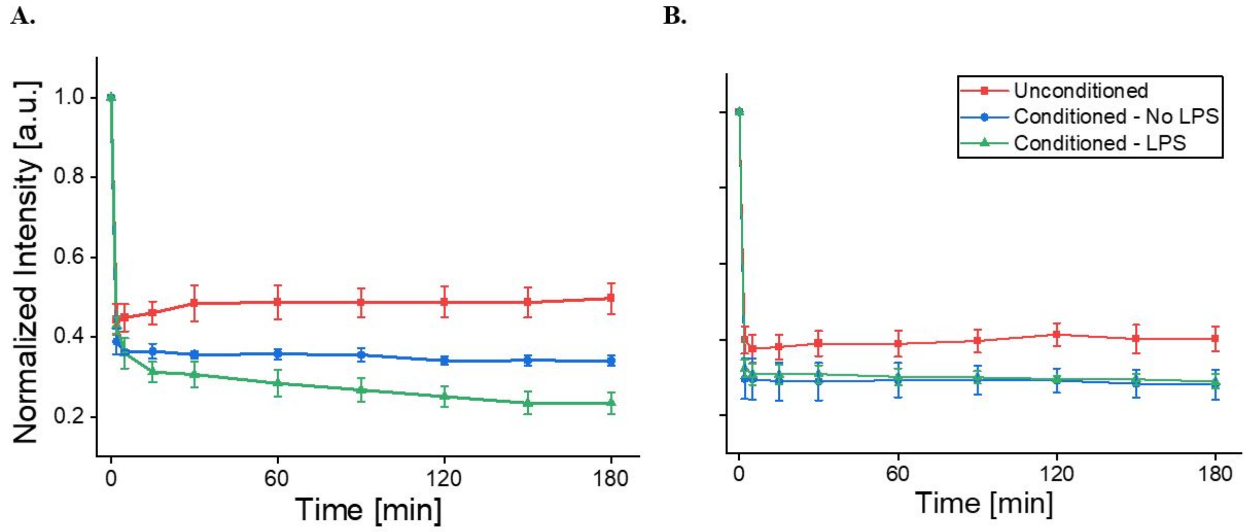

Figure S6. Time-sensitive response of SWCNT-ssDNA to conditioned media from Raw 264.7 cells. A) (7,5) Fluorescence intensity of SWCNT-31Apt in response to cell media samples over three hours. B) (7,5) Fluorescence intensity of SWCNT-$(GT)_{15}$ in response to cell media samples over three hours (n = 3; mean ± standard deviation).
